# Use of mobile phones and text messaging to decrease the turnaround time for early infant HIV diagnosis and notification in rural Zambia: an observational study

**DOI:** 10.1186/s12887-017-0822-z

**Published:** 2017-03-08

**Authors:** Catherine G. Sutcliffe, Philip E. Thuma, Janneke H. van Dijk, Kathy Sinywimaanzi, Sydney Mweetwa, Mutinta Hamahuwa, William J. Moss

**Affiliations:** 1 Department of Epidemiology, Bloomberg School of Public Health, Johns Hopkins University, 615 North Wolfe Street, Baltimore, MD USA; 2 Macha Research Trust, Macha Hospital, Choma, Zambia; 3 Department of Immunology and Infectious Diseases, Erasmus University Rotterdam, Rotterdam, The Netherlands

**Keywords:** mhealth, HIV, Infant diagnosis, Sub-Saharan Africa

## Abstract

**Background:**

Early infant diagnosis of HIV infection is challenging in rural sub-Saharan Africa as blood samples are sent to central laboratories for HIV DNA testing, leading to delays in diagnosis and treatment initiation. Simple technologies to rapidly deliver results to clinics and notify mothers of test results would decrease many of these delays. The feasibility of using mobile phones to contact mothers was evaluated. In addition, the first two years of implementation of a national short message service (SMS) reporting system to deliver test results from the laboratory to the clinic were evaluated.

**Methods:**

The study was conducted in Macha, Zambia from 2013 to 2015 among mothers of HIV-exposed infants. Mothers were interviewed about mobile phone use and willingness to be contacted directly or through their rural health center. Mothers were contacted according to their preferred method of communication when test results were available. Mothers of positive infants were asked to return to the clinic as soon as possible. Dates of sample collection, delivery of test results to the clinic and notification of mothers were documented in addition to test results.

**Results:**

Four hundred nineteen mothers and infants were enrolled. Only 30% of mothers had ever used a mobile phone. 96% of mobile phone owners were reached by study staff and 98% of mothers without mobile phones were contacted through their rural health center. Turnaround times for mothers of positive infants were approximately 2 weeks shorter than for mothers of negative infants. Delivery of test results by the national SMS system improved from 2013 to 2014, with increases in the availability of texted results (38 vs. 91%) and arrival of the texted result prior to the hardcopy report (27 vs. 83%). Texted results arriving at the clinic before the hardcopy were received a median of 19 days earlier. Four discrepancies between texted and hardcopy results were identified out of 340 tests.

**Conclusions:**

Mobile phone and text messaging technology has the potential to improve early infant diagnosis but challenges to widespread implementation need to be addressed, including low mobile phone ownership, use and coverage in rural areas.

## Background

Over 90% of the world’s 2.9 million HIV-infected children reside in sub-Saharan Africa where the resources for providing care and treatment are lowest [1]. Over the past decade, the capacity for providing antiretroviral treatment to HIV-infected individuals has increased dramatically, with an estimated 630,000 children in sub-Saharan Africa receiving treatment at the end of 2013 [1]. This represents, however, only 22% of children living with HIV in need of antiretroviral therapy, compared to an estimated coverage of 37% among all people living with HIV [1]. One of the main factors for low coverage among children is the large number of infants who remain undiagnosed but would be eligible for treatment under current treatment guidelines.

Diagnosis of HIV infection in early infancy is difficult in sub-Saharan Africa, as assays based on HIV DNA or RNA detection must be used rather than the serologic assays used in older children and adults [2]. Unfortunately, these assays require a high level of technology, training and cost, and are primarily available in major urban areas. Consequently, HIV clinics in rural areas, where many HIV-exposed and infected infants reside, must transport specimens to central laboratories. This can result in a delay in diagnosis as specimens and test results must be transported from and to the HIV clinics and then disclosed to the mother at their next clinic appointment. The World Health Organization recommends that results be disclosed to mothers within 4 weeks of specimen collection [3]. Data are emerging, however, that this process takes longer than 4 weeks in many rural areas of sub-Saharan Africa [4–6]. In rural Zambia, the median time from sample collection to disclosure of results to the mother was 92 days and delays were identified in returning results from the lab to the clinic and from the clinic to the caregiver [7].

Mobile phones have recently become more widely available in sub-Saharan Africa and are a promising tool for improving outcomes for many areas related to clinical care and treatment of HIV infection and other conditions. Mobile phones have been successfully used in low-resource healthcare settings to deliver HIV test results from central laboratories to rural clinics [5, 6, 8, 9], notify and remind patients of upcoming or missed appointments to increase retention [10–12], and as a reminder system for HIV-infected individuals receiving treatment to increase adherence [13, 14]. Simple technologies to more rapidly deliver test results to clinics and notify mothers of the availability of test results would decrease much of the delay associated with the current HIV testing strategy in infants.

The objective of this study was to evaluate the use of mobile phones in rural Zambia to improve the process of early infant diagnosis. The feasibility of using mobile phones to notify mothers of the availability of test results either directly or through their local rural health center was assessed. In addition, the performance of a national short message service (SMS) reporting system to deliver test results from the central laboratory to the clinic was evaluated during the first two years of implementation in rural Zambia.

## Methods

### Ethics statement

This study was approved by the Government of Zambia Ministry of Health and the Institutional Review Boards at the Johns Hopkins Bloomberg School of Public Health and Macha Research Trust. Written informed consent was obtained from the parent or caregiver for participation in the study.

### Study setting

The study was conducted at Macha Hospital in a rural area of Choma District, Southern Province, Zambia. The catchment area of Macha Hospital is populated by subsistence farmers living in small, scattered homesteads. Macha Hospital is administered by the Brethren in Christ Church but functions within the healthcare system of the Ministry of Health. The hospital serves as a district-level referral hospital for rural health centers within an 80 kilometer radius, serving a population of over 150,000 persons.

Since 2005, Macha Hospital has operated an HIV clinic providing programs for the prevention of maternal-to-child HIV transmission (PMTCT) and for the care and treatment of HIV-infected individuals [15, 16]. Since 2008, early infant diagnosis has been available with HIV DNA testing performed at a central laboratory in Lusaka. HIV-infected pregnant women and infants receive care and treatment according to Ministry of Health [17] and WHO guidelines [18].

The current recommended schedule for early infant diagnosis in Zambia is HIV DNA testing at 6 weeks and 6 months of age, with confirmatory testing of all positive results. Serologic testing is recommended at 12 and 18 months of age and 2 months after weaning, with confirmatory HIV DNA testing of any positive results [17]. When infants are brought to either the HIV clinic or a primary health center associated with the hospital for early infant diagnosis, a dried blood spot (DBS) card is collected and processed. Mothers are typically provided with an appointment 3 months later to be informed of the test results. Approximately once per month, DBS cards are transported to Lusaka (~350 km by road) for testing, and results from previous batches are collected and transported back to the clinic.

Due to the long turnaround times for clinics and mothers to receive test results, an SMS reporting system was successfully piloted in Zambia to facilitate reporting of test results from the central laboratory to the clinic [9]. The SMS reporting system was subsequently implemented by the government as part of the national HIV program at Macha Hospital in January 2013. When testing is completed at the central lab and results are available, an automated text message is sent to a designated healthcare worker at the clinic to prompt them to retrieve results from the system. When the healthcare worker responds by text with their unique identifying number, all available results are texted back through the automated system.

### Study procedures

This study was nested within an ongoing study of early infant diagnosis that began at Macha Hospital on April 1, 2013. Mothers bringing infants to either the HIV clinic or a primary health center associated with the hospital were eligible for enrollment. After enrollment, a questionnaire was administered by study staff to collect information on demographics, antenatal care, prior HIV testing, and mobile phone use. Mothers who reported access to a mobile phone were asked if they consented to being contacted by study staff when their child’s test results were available and, if willing, were asked to provide a phone number. All mothers, regardless of whether they had access to a mobile phone, were asked if they consented to study staff contacting their local rural health center when their child’s test results were available, and if willing, were asked to name their local rural health center and provide the name of their village. Information on use of antiretroviral drugs for the prevention of mother-to-child transmission was abstracted from the mother’s medical record.

As part of routine clinical care, a DBS card was collected from the HIV-exposed infant and sent to Lusaka for testing. Study staff were alerted by clinic staff when the result was delivered to the clinic either by SMS or hard copy. The test result and testing dates were abstracted from the hardcopy result returned to the clinic. The dates the results were delivered to the clinic by SMS and hard copy were documented by study staff either from communication with clinic staff or from the national DBS register at the clinic.

As part of the study, mothers who consented to be contacted by mobile phone were called or texted (in the local language), depending on their preference, by study staff when the results were available. Due to discrepancies found between SMS and hardcopy results for two tests in February 2014, the study protocol was changed in March 2014 to notify mothers upon receipt of the hardcopy results, which were assumed to be the true result. Mothers were told that the results were available but the test result was not provided. Mothers of infants with detectable HIV DNA were told to return to the clinic as soon as possible. Mothers of infants with undetectable HIV DNA were told to return to the clinic for their regularly scheduled appointment.

For mothers without access to mobile phones who consented to be contacted, study staff communicated with the healthcare worker at the local rural health center by mobile phone. They were told that the test results were available and asked to notify the mother. Mothers were typically contacted through the network of community health workers at the health centers. Community health workers are usually volunteers from the community and they made home visits to notify the mother to return to the clinic for their results. Study staff documented all contact attempts and outcomes of contacts with both mothers and healthcare workers at the rural health centers. They were instructed to call as many as three times if necessary and to vary the time and day of the call if they were not successful with the first attempt. Study staff also monitored clinic and medical records to document when the mother returned to receive their child’s test results.

### Statistical analysis

This study included all mothers enrolled between April 1, 2013 and March 31, 2015. Descriptive statistics were used for the analysis. Chi-square tests were used to compare categorical variables and Wilcoxon rank sum tests were used to compare continuous variables. From April 1, 2013 to March 15, 2014, the date that test results were delivered to the clinic was assumed to be either the date the SMS result was received or the date the hardcopy result was received, whichever came first. From March 16, 2014 to March 31, 2015, the date that test results were delivered to the clinic was assumed to be the date the hardcopy result was received.

## Results

Between April 1, 2013 and March 31, 2015, 594 tests for early infant diagnosis were conducted among 419 infants and included in the analysis, representing >95% of tests conducted at the clinics during this period. Half of all infants were male (52%) and the median age of the first test during the study period was 8.7 weeks (interquartile range [IQR]: 6.0, 26.1) (Table 1). All infants were brought for testing by their mothers and only 3 (1%) reported that the father had died. The median age of the mothers was 32 years (IQR: 26, 35) and the majority (71%) had at most completed primary school.

**Table 1:** Characteristics of HIV-exposed infants and their caregivers at the first test in rural Zambia

Infant	*N* = 419
Male: n (%)	217 (52)
Age (weeks): median (IQR)	8.7 (6.0, 26.1)
Parent/guardian	*N* = 419
Relationship to child - mother: n (%)	419 (100)
Age (years): median (IQR)	32 (26, 35)
Education: n (%)	
None	22 (5)
Primary (1–7)	275 (66)
Secondary (8–12)	113 (27)
University/college/certificate	9 (2)
Transportation to the clinic^a^: n (%)	
Walked	121 (29)
Bicycle	84 (20)
Public or group transport	156 (37)
Car	50 (12)
Motorcycle	9 (2)
Travel time to the clinic (hours): n (%)	
< 1	27 (6)
1–2	92 (22)
3–4	192 (46)
5+	108 (26)
Travel costs (ZMW)^b^: n (%)	
1–10	6 (1)
11–20	34 (8)
21–30	65 (16)
31–40	58 (14)
> 40	28 (7)
None	228 (54)

^a^ Categories not mutually exclusive (2 people reported multiple forms of transportation; 1 person did not respond)

^b^ 1 US dollar (USD) ~ 5 Zambian Kwacha (ZMW)

### Mobile phone use

Only 30% of mothers reported ever using a mobile phone and reported use was similar over time (2013: 33%; 2014: 26%; 2015: 30%). Ever users of a mobile phone were more likely to have completed secondary school or higher training than never users (45% vs. 22%; *p* < 0.0001), but were of similar age (median 30 vs. 32 years; *p* = 0.45). Among mothers reporting ever using a phone, 74% had ever sent and 90% had ever received a text message and 94% owned their own phone.

**Fig. 1:**
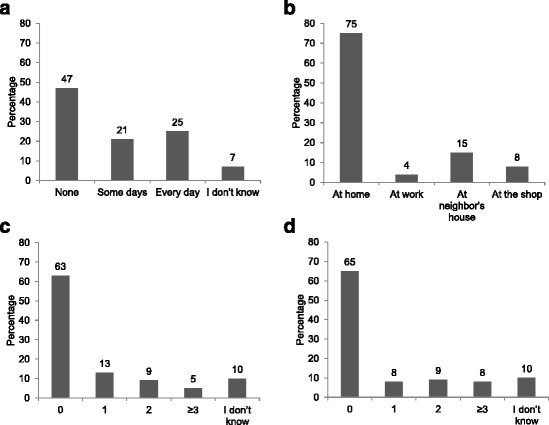
**a** Mobile phone coverage in the home during the past week; **b** Location for charging phones (categories are not mutually exclusive; only 2% of mothers reported charging their phone in multiple locations); **c** Number of times the phone lacked power in the past week due to inability to complete charging; **d** Number of days in the past week the phone was not fully functional (functional was defined as having both a signal and sufficient battery to make a call or send or receive an SMS message)

Among the 119 phone owners, the majority (92%) owned only one phone. Among the seven ever users who did not own a phone, 29% borrowed their spouse’s phone. Cell phone network providers included Airtel (88%), MTN (18%), and Zamtel (13%). Nineteen percent of mothers reported using multiple providers, as many phones are capable of carrying two subscriber identification module (SIM) cards. Approximately half (46%) of mothers reported having cell phone coverage in their homes in the past week and the majority (75%) were able to charge their phones at home (Fig. 1). Most mothers (65%) reported having a fully functional phone (signal and battery) on all days during the prior week (Fig. 1). Twenty-eight percent of mothers reported sharing their phone with someone else, primarily their spouse (82%) but also their children (12%), other adults in the household (3%), or relatives outside the household (3%). Adults in the household, but not necessarily the children, were aware of their HIV infection status. The majority of mothers (87%) were able to read Chitonga (the local language) and thus could read text messages.

### Notifying mothers of availability of test results using mobile phones

Of the 119 mothers who owned a mobile phone, 99% agreed to be contacted. One person declined due to concerns about confidentiality. Eighty-seven percent of mothers agreeing to be contacted preferred a phone call rather than a text message, and 95% provided a phone number at the study visit.

**Fig. 2:**
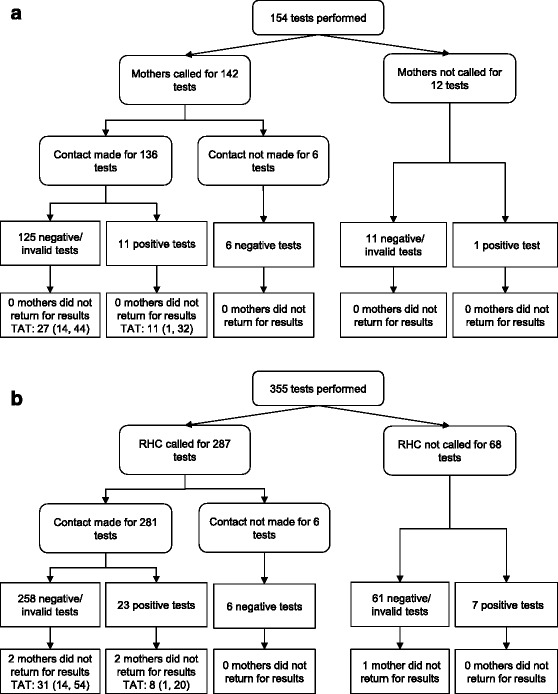
Flow chart for mothers contacted directly by mobile phone (**a**) or indirectly through their rural health center (**b**) in rural Zambia. *RHC*: rural health center. *TAT*: turnaround time; median (IQR) days from notifying mothers of availability of results to disclosure of results

HIV DNA test results were available for 154 tests performed from April 1, 2013 to December 1, 2014 for 101 infants whose mothers agreed to be contacted by phone and provided a phone number (Fig. 2). Twelve mothers were inadvertently not called due to errors by study staff. Eighty-nine mothers were called for 142 test results. Contact was made with the mother for 96% of tests. The remaining 4% of mothers were out of the coverage area for all call attempts made by study staff and contact was not made. One phone call was made for 81% of tests, 2 calls for 14%, 3 calls for 4%, and 4 calls for 2% of tests. Mothers were first called a median of 6 days (IQR: 4, 14) after the test results were received at the clinic.

All mothers returned for their child’s results (Fig. 2). The median time from delivery of the test results to the clinic to disclosure of results to the mother was 35 days (IQR: 20, 54). This period of time was shorter for mothers of infants with positive results who were told to return to the clinic as soon as possible (median: 18 days; IQR: 5, 40) than for mothers of infants with negative or invalid results who were told to attend their next scheduled visit (median: 35 days; IQR: 21, 54)(*p* = 0.11). Three infants, all with positive tests, died before the mother returned for results.

Of the 12 positive tests, five occurred among infants receiving a first positive result during the study period and surviving past diagnosis. One infant did not return to initiate ART. The median time from sample collection to ART initiation among the other four infants was 103 days (IQR: 71, 144).

### Contacting rural health centers to notify mothers of availability of test results using mobile phones

All mothers were asked if they would agree to study staff contacting their rural health center when test results were available and 413 (99%) agreed. The primary reason for refusal was concern about confidentiality at the rural health center.

HIV DNA test results were available for 355 tests performed from April 1, 2013 to December 1, 2014 for 254 infants whose mothers agreed to have the health center contacted by phone (Fig. 2). Calls were not made for 40 mothers due to errors on the part of study staff and for 28 mothers as a phone number for the healthcare worker at the rural health center was never identified. Calls were made to the health center to contact 193 mothers for 287 test results. Contact was made with a healthcare worker for 98% of tests. The number of phone call attempts to make contact was one for 78% of tests, 2 for 18%, 3 for 4%, and 4 for 0.3% of tests. Rural health centers were first called a median of 7 days (IQR: 3, 20) after the test results were received at the clinic.

Five mothers, four of whom had been contacted, did not return for their child’s results (Fig. 2). The median time from delivery of test results to the clinic to disclosure of results to the mother was 37 days (IQR: 17, 57). This period of time was shorter for mothers of infants with positive results who were told to return to the clinic as soon as possible (median: 15 days; IQR: 7, 27) than for mothers of infants with negative or invalid results who were told to attend their next scheduled visit (median: 39 days; IQR: 18, 61) (*p* < 0.0001). Two children, both of whom tested positive, died before the mother returned for results.

Of the 30 positive tests, 15 occurred among infants receiving a first positive result during the study period and surviving past diagnosis and whose mothers returned for their test results. One infant did not return to initiate ART. The median time from sample collection to ART initiation among the other 14 infants was 57 days (IQR: 35, 75).

### National SMS reporting system to deliver test results to the clinic

HIV DNA results were available in hardcopy for 510 tests. Only 340 (67%) hardcopy results were accompanied by a result reported through the national SMS reporting system. SMS reporting improved over the duration of the study, with 38 and 91% of texted results available for HIV DNA tests performed in 2013 and 2014, respectively.

Of the 340 tests with texted results, only 231 (68%) texted results arrived at the clinic prior to the hardcopy result. This also improved over time, with 27 and 83% of results arriving prior to the hardcopy in 2013 and 2014, respectively. The 231 texted results arrived at the clinic a median of 19 days (IQR: 10, 25; range: 1, 82) before the hardcopy result, resulting in a median turnaround time from the date the sample was collected to delivery of the results to the clinic of 36 days (IQR: 26, 45; range: 12, 70) compared to 53 days (IQR: 43, 68; range: 22, 134) for the corresponding hardcopy results. The 109 texted results that arrived at the same time or after the hardcopy result arrived a median of 11 days later (IQR: 8, 13; range: 0, 49).

During the study period, four discrepancies (1%) were identified (two in February, one in April and one in July, 2014) between the result reported on the hardcopy and the result reported by SMS (two for which the text message was positive and hardcopy negative and two for which the text message was negative and hardcopy positive). For the first two, the mother was notified of the initial texted result and had to be recontacted after the discrepancy was discovered.

## Discussion

In this study in rural Zambia, mobile phone use by HIV-infected mothers was low but mobile phones and text messaging had the potential to impact turnaround times for early infant diagnosis. The majority of mothers agreed to be contacted either directly or through their rural health centers and most mothers were successfully contacted when test results were available. Mothers of infected infants returned to the clinic in a significantly shorter time, leading to earlier diagnosis and enrollment into care. In addition, the SMS reporting system at the central lab successfully reduced the turnaround time for returning test results to the clinic when functioning properly.

Contacting mothers of infected infants either directly or through their rural health center succeeded in reducing the time between when test results were delivered to the clinic and when results were disclosed to the mother by approximately 50%. Mothers were provided the test results more than 2 weeks earlier, providing an earlier opportunity for the child to be enrolled into HIV care and to initiate antiretroviral therapy. While the numbers are small, the time to initiate ART in this study (median: 66 days combining mothers contacted directly or through the clinics) was shorter than previously reported from this clinic (median: 101 days) [7]. Early initiation of treatment has been shown to reduce HIV-associated morbidity and mortality [19] and has the potential to limit damage to the developing immune system [20].

Mothers of uninfected infants were also contacted to notify them that results were available and that they should attend their regularly scheduled appointments. Almost all mothers returned for their child’s results. While a comparison group was not available, previous data from the same clinic indicated that approximately 7% of mothers failed to return for test results [7]. Use of mobile phones has been shown in other settings and programs to increase retention in HIV care [10–12].

Implementing an intervention based on mobile phones was not without challenges in this setting. First, mobile phone use in this rural community was lower than anticipated and many mothers who did own a phone did not know their number or bring the phone with them. While access to mobile phones has expanded dramatically throughout sub-Saharan Africa in the past decade, there is still heterogeneity of access and use within and between countries [21]. A recent survey found that 78% of Zambians owned a phone, similar to other countries in sub-Saharan Africa [22]. However, ownership was lower among those with lower education and income and those residing in rural areas. Strategies to improve turnaround times based on direct communication with mothers in rural communities using mobile phones may not currently be feasible. In contrast, healthcare workers are likely to have mobile phones and the network of community health workers and volunteers at rural health centers and health posts could be used to reach mothers.

Second, contacting mothers indirectly through healthcare workers was not always possible as a clinic phone number could not be found for 10% of participants. These clinics tended to be in other districts and further from Macha. The system worked best for rural health centers in the immediate catchment area of the hospital.

Third, coverage of mobile providers was unreliable. For 20% of tests, more than one call had to be made to either the mother or the healthcare worker and contact was never made for 3% of tests because the mother or healthcare worker was out of the coverage area. This increased the burden on study staff who had to make multiple call attempts and track all of their calls and delayed contact with the mother.

Lastly, implementing this intervention required additional resources, including staff dedicated to tracking and monitoring results and contacting mothers, a study mobile phone, and monthly purchase of ‘talk time’ (total <50 USD per month) for the study mobile phone. Involving healthcare workers at the rural health centers increased the burden on an already busy and understaffed system. While it may not be feasible for programs to implement this system for all mothers, depending on the number of infants tested, it should be feasible to focus on contacting mothers of infected infants and ensuring that HIV-infected infants are diagnosed early, retained in care, and started on treatment.

A national SMS reporting system was also implemented at the clinic as part of the government HIV program during the study period after being successfully evaluated in a nearby region [9]. In a pilot study conducted by other investigators, the time between sample collection and delivery of results to the clinic was reduced from 44 to 27 days [9]. Similar reductions were observed in other programs using cellular-based systems for transmitting results [23, 24]. The first year of implementation of the text message system was challenging, with most test results either not texted or sent after the hardcopy result was available. Implementation improved in the second year, with results texted to the clinic almost 3 weeks prior to receipt of the hard copy. Unfortunately, discrepancies were found between the texted and hardcopy results in 1% of tests and the mother was provided the incorrect diagnosis. These discrepancies caused staff to not trust the text message system and they subsequently preferred to wait for hardcopy results before notifying the mother. Training staff involved in the text message system at the central lab should emphasize the purpose and importance of sending accurate and prompt messages to increase understanding and compliance. In addition, quality control measures, such as required confirmation prior to sending SMS texts with positive results and regular monitoring at the lab for discrepancies in SMS and hardcopy results, should be put in place to improve implementation of text message systems.

There were several limitations to this study. First, the evaluation of text messages to notify mothers of the availability of test results was a feasibility study and the intervention was provided to all participants. Consequently, there was no control group and it was not possible to determine the impact of the intervention on retention. Second, the evaluation of the national SMS system was performed at the clinics and did not involve the central laboratory or the individuals involved in sending the text messages. We therefore do not know how they were trained, the reasons for the delays in sending the text messages, or the quality control procedures in place for identifying and preventing discrepancies. Third, due to the discrepancies identified, the hardcopy results were disclosed to the mother. As a result, we were not able to determine the full benefit of mobile technology in reducing the turnaround time from the lab to the clinic. Lastly, this study was conducted in one rural region of Zambia and may not be representative of other regions of Zambia or sub-Saharan Africa. However, the challenges faced are similar to those in other rural areas and we anticipate that both the benefits and challenges of using mobile technology may also be similar.

## Conclusions

Mobile phones have the potential to improve turnaround times and overcome many challenges in implementing early infant diagnosis in rural areas. When used in combination, text message systems for delivering results from the lab to the clinic and mobile phones for notifying mothers directly or through their clinics could reduce the time to diagnose HIV-infected children by over 1 month and enable earlier initiation of HIV care and treatment. Implementation of these systems, however, is challenging and requires significant human and financial resources. Additional resources are also necessary for ongoing monitoring to ensure quality. Until a point-of-care test is available for early infant diagnosis, programs and clinics will need to weigh the costs of text message systems against the benefits of earlier diagnosis and treatment for HIV-infected infants.

## Abbreviations

DBS: Dried blood spot
DNA: Deoxyribonucleic acid
HIV: Human immunodeficiency virus
IQR: Interquartile range
PMTCT: Prevention of mother-to-child transmission
RNA: Ribonucleic acid
SMS: Short message service
USD: United States dollar
ZMW: Zambian kwacha
